# A novel prognostic model for adult patients with Hemophagocytic Lymphohistiocytosis

**DOI:** 10.1186/s13023-020-01496-4

**Published:** 2020-08-20

**Authors:** Jun Zhou, Jing Zhou, Zhi-Qi Wu, Hemant Goyal, Hua-Guo Xu

**Affiliations:** 1 Department of Laboratory Medicine, the First Affiliated Hospital of Nanjing Medical University, Nanjing, Jiangsu China; 2 The Wright Center of Graduate Medical Education, Scranton, USA

**Keywords:** Hemophagocytic Lymphohistiocytosis, Model, Prognosis, Ferritin, Overall survival

## Abstract

**Background:**

Hemophagocytic Lymphohistiocytosis (HLH) is a type of rare disease with low survival rate. We aimed to develop a model to evaluate the six-month prognosis in adult HLH patients. The data at discharge (will be called as post-treatment) for newly diagnosed adult HLH patients was collected and independent prognostic variables were selected for inclusion in the model.

**Results:**

Three laboratory markers were confirmed to be the independent risk factors (ferritin: hazard ratio (HR) 0.101, 95% confidence interval (CI) 0.036–0.282, *P*<0.001; platelets: HR 4.799, 95% CI 1.884–12.223, *P* = 0.001; alanine aminotransferase (ALT): HR 0.423, 95% CI 0.180–0.997, *P* = 0.049). These were included in the final clinical prediction model. Receiver operating characteristic (ROC) curves disclosed that this model had a better discrimination (area under the curve (AUC) = 0.842, 95% CI 0.773–0.910, *P* < 0.001) than each of them alone and the calibration curves aligned completely with the model predictions and actual observations. Kaplan-Meier curves revealed a significant difference in the overall survival (OS) in patients stratified by the model with higher values associated with a better OS.

**Conclusion:**

These results point out that serum ferritin, platelets and ALT levels are independent elements of OS in adult patients with HLH, and that the proposed model have a better prognostic value than any of these markers alone.

## Background

Hemophagocytic Lymphohistiocytosis (HLH) is a rare invariable fatal disease (if untreated) accompanied with the secretion of a huge number of cytokines which then trigger a series of chain inflammatory reactions [1, 2]. Though clinical manifestations of HLH vary, fever (peak > 38.5 °C), hepatomegaly, splenomegaly, jaundice, edema, cytopenia, hypertriglyceridemia, hypofibrinogenemia, hyperferritinemia and hemophagocytosis are the major performance [3–5]. HLH is made up by congenital and acquired disease with the former one associated with family heredity or identifiable gene mutations [6] and the other one is usually due to infectious diseases, immune dysfunction, acquired immune deficiency, or malignancies [7, 8]. However, some genetic abnormalities can also be found in the acquired HLH [9, 10].

In the past most of guidelines (diagnostic, treatment and prognostic), meta-analysis and clinical trials regarding to HLH were summed up from children [11]. However, greater number of adult HLH cases are being diagnosed and reported because of increasing recognition and better management [12]. In Japan, A nationwide study presented that adults of HLH accounts for 40% of HLH [13]. The adult HLH progression is usually complicated, sometimes changeable and the mortality rate varies considerably from 8 to 60% [14–17]. Multiorgan failure, hemorrhage, or sepsis are the main reason for mortality in HLH which can be managed appropriately if recognized early [17]. Therefore, to discern the patients who are in danger of poor prognosis is crucial in adult HLH. Although, some studies have reported few prognostic factors in adult HLH [18, 19], there is no single effective indicator because of low sensitivity or specificity. Thus, a clinical prediction model based on the clinical and laboratory data may support medicals to grasp precise evaluation of patients’ prognosis.

Clinical models, a combination of all or most independent risk factors progression of the disease, could apply to compute and forecast incidents occurrence probability. More and more models are being widely established to aid in identifying patients who are at high risk for events in several diseases such as colon carcinoma and autoimmune thyroid disease [20, 21]. As far as we know, there are few models available for predicting the risk of early death in adult HLH patients. Here we establish a model to assess the prognosis within 6 months in adult HLH patients.

## Results

### The characteristics of adult HLH patients

There are 136 adult HLH patients (78 males, 58 females) including in the study. 94, 25, 13 and 4 of patients were positive for 5/8, 6/8, 7/8, or 8/8 HLH criteria. The underlying etiologies included infectious diseases (*N* = 42), malignancies (*N* = 38), autoimmune disorders (*N* = 3), multiple etiologies (*N* = 26) and no identified underlying disorder (*N* = 27). Supplement Table 1 exhibits the admission (will be called as pre-treatment) demographic, clinical characteristics and laboratory data of adult HLH patients. No remarkable difference was noticed in the clinical and laboratory data (except direct bilirubin (DBIL), albumin, urea nitrogen (UREA) and creatinine (CREA)) between the two groups (all P > 0.05). However, the data at discharge (will be called as post-treatment) showed the levels of hemoglobin (HB), platelets, albumin and calcium (Ca^2+^) in the survivor group were markedly elevated than those in non-survivor group (all *P* < 0.05); While ferritin, aspartate aminotransferase (AST), lactate dehydrogenase (LDH), α-hydroxybutyrate dehydrogenase (α-HBDH), DBIL, UREA, CREA and uric acid (UA) in the survivor group were obvious lower than that in non-survivor group (all *P* <0.05) (Table 1). Chemotherapy or other cytotoxic drugs such as cyclophosphamide or etoposide were used in 77 patients; 59 of the study patients received symptomatic treatment (methotrexate, steroids and supportive care). In addition, in non-survivor group, 43 patients received symptomatic treatment and 28 patients received chemotherapy or other cytotoxic drugs; 34 patients received symptomatic treatment and 31 patients received chemotherapy or other cytotoxic drugs in survivor group.

**Table 1:** Post-treatment clinical characteristics of patients according to outcome

Characteristics	Survivors, *n* = 65	Nonsurvivors, *n* = 71	*P*-value
**Gender (male/female), n**	31/34	47/24	0.066
**Median age (range), y**	48 (18–78)	52 (18–78)	0.104
**Ferritin (μg/L)**	933.1 (45.3, > 15,000)	3740 (250, > 15,000)	< 0.001
**FIB (g/L)**	2.29 ± 1.05	1.95 ± 1.04	0.113
**Neutrophils (×10**^**9**^**/L)**	3.80 ± 3.06	3.12 ± 3.76	0.253
**HB (g/L)**	99.88 ± 18.58	88.41 ± 19.26	0.001
**Platelet (×10**^**9**^**/L)**	118.51 ± 75.10	55.79 ± 66.02	< 0.001
**ALT (U/L)**	90.8 ± 131.36	200.61 ± 449.48	0.060
**AST (U/L)**	84.39 ± 154.33	302.64 ± 562.74	0.003
**LDH (U/L)**	420.99 ± 330.20	1561.83 ± 2604.34	0.001
**α-HBDH (U/L)**	270.17 ± 178.31	780.96 ± 1095.77	< 0.001
**DB (μmol/L)**	7.83 ± 9.41	35.09 ± 51.60	< 0.001
**TG (mmol/L)**	2.02 ± 1.16	4.14 ± 8.59	0.051
**HDL (mmol/L)**	1.08 ± 0.45	0.94 ± 2.09	0.589
**LDL (mmol/L)**	2.73 ± 1.20	2.64 ± 3.26	0.883
**Albumin (g/L)**	33.16 ± 5.35	27.50 ± 5.01	< 0.001
**Glucose (mmol/L)**	5.67 ± 2.58	6.19 ± 2.77	0.262
**UREA (mmol/L)**	5.84 ± 2.57	10.81 ± 9.05	< 0.001
**CREA (μmol/L)**	53.42 ± 38.57	94.80 ± 115.76	0.007
**UA (μmol/L)**	218.61 ± 97.35	311.69 ± 229.97	0.003
**Ca**^**2+**^ **(mmol/L)**	2.15 ± 0.16	2.02 ± 0.19	< 0.001

*ALT* alanine aminotransferase, *AST* aspartate aminotransferase, *Ca*^*2+*^ calcium, *CREA* creatinine, *DBIL* direct bilirubin, *FIB* fibrinogen, *HB* hemoglobin, *α-HBDH* hydroxybutyrate dehydrogenase, *HDL* high density lipoprotein, *LDH* lactate dehydrogenase, *LDL* low density lipoprotein, *TG* triglycerides, *UREA* urea nitrogen

### Independent significant factors in the cohort

To further identify the six-month prognostic factors, we evaluated the post-treatment laboratory data as categorical variables by the logistic regression method. We first used univariate logistic regression method to differentiate adverse elements (dichotomous variables) (Table 2). The results indicated that patients’ ferritin, neutrophils, HB, platelets, alanine aminotransferase (ALT), AST, LDH, α-HBDH, DBIL, albumin, UREA, CREA, UA and Ca^2+^ were associated with the six-month prognosis in HLH patients (all *P*<0.05). Then a multivariate method was performed to verify whether the factors were independent risk factors. As a result, three variables were confirmed to be the independent prognostic factors: ferritin: hazard ratio (HR) 0.101, 95% confidence interval (CI) 0.036–0.282, *P*<0.001; platelets: HR 4.799, 95% CI 1.884–12.223, *P* = 0.001; ALT: HR 0.423, 95% CI 0.180–0.997, *P* = 0.049 (Table 2).

**Table 2:** Univariate and multivariate analyses of factors which affect the outcome of HLH

Variables	Univariate analysis			Multivariate analysis			
	HR	95% CI	*P* value	β	HR	95% CI	*P*-value
**Ferritin**	0.070	0.026–0.184	< 0.001	−2.290	0.101	0.036–0.282	< 0.001
**Neutrophils**	3.414	1.400–8.325	0.007				
**HB**	3.251	1.577–6.703	0.002				
**Platelets**	6.364	2.840–14.260	< 0.001	1.568	4.799	1.884–12.223	0.001
**ALT**	0.298	0.147–0.607	0.001	−0.860	0.423	0.180–0.997	0.049
**AST**	0.409	0.203–0.824	0.015				
**LDH**	0.321	0.147–0.699	0.005				
**α-HBDH**	0.231	0.101–0.530	< 0.001				
**DBIL**	0.262	0.129–0.543	< 0.001				
**Albumin**	9.684	1.176–79.738	0.010				
**UREA**	0.235	0.103–0.533	< 0.001				
**CREA**	0.085	0.011–0.680	0.001				
**UA**	0.334	0.136–0.821	0.020				
**Ca**^**2+**^	4.593	1.949–10.824	< 0.001				
**AUC**		0.842					

*ALT* alanine aminotransferase, *AUC* area under the curve, *CA* calcium, *CREA* creatinine, *CI* confidence interval, *DBIL* direct bilirubin, *HB* hemoglobin, *α-HBDH* hydroxybutyrate dehydrogenase, *HR* hazard ratio, *LDL* low density lipoprotein, *UREA* urea nitrogen, *β* regression coefcient

**Fig. 1:**
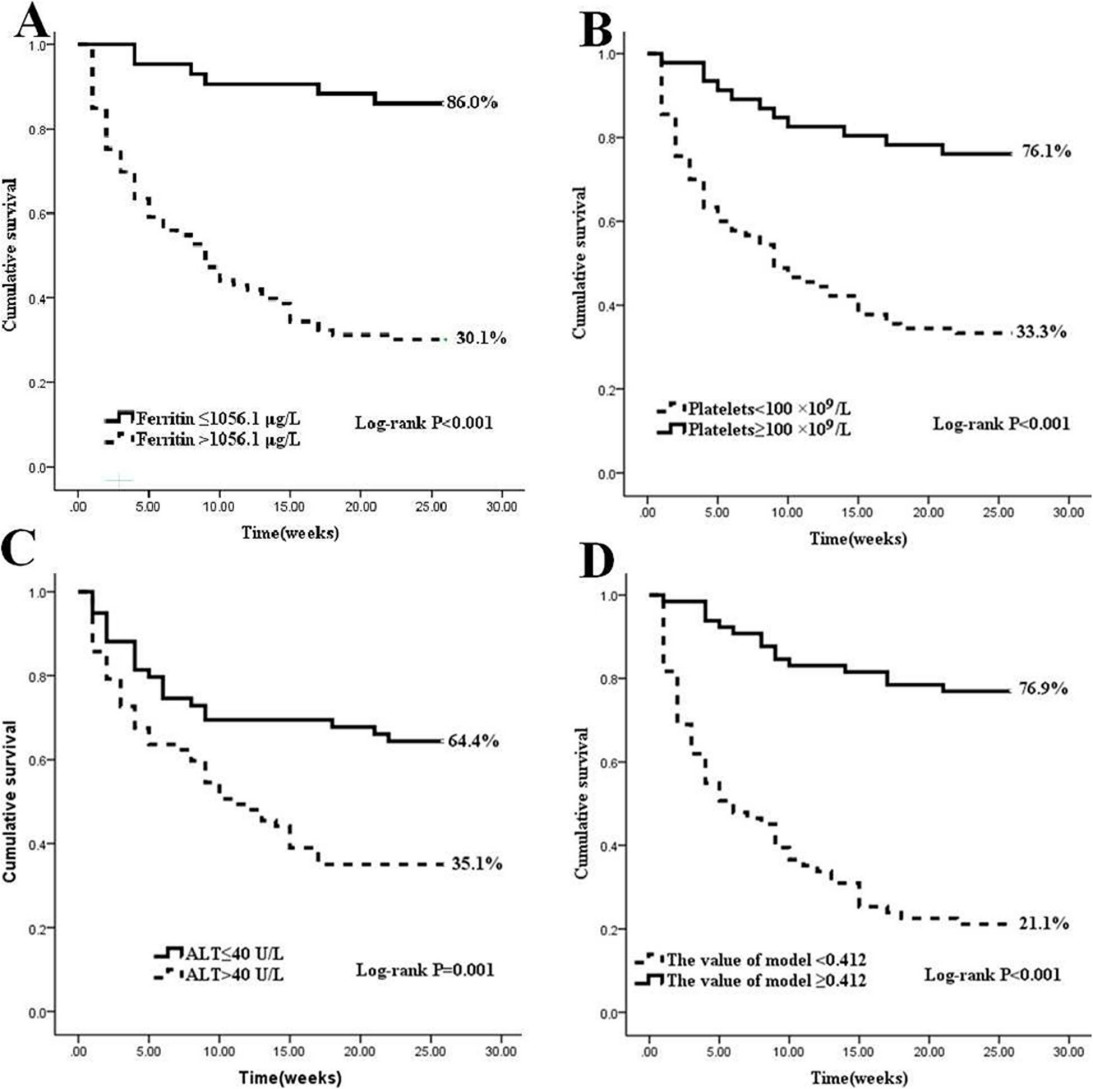
Survival curves of HLH patients with high risk vs. low risk of early death. **a** Post-treatment serum ferritin level > 1056.1 μg/L was associated with significantly worse OS than those with ≤1056.1 μg/L. **b** Post-treatment serum platelets < 100 × 10^9^/L showed significantly worse OS than those with post-treatment serum platelets ≥100 × 10^9^/L. **c** Post-treatment serum ALT > 40 U/L showed significantly worse OS than those with post-treatment serum ALT ≤40 U/L. **d** The value of model < 0.412 showed significantly worse OS than those with The value of model ≥0.412

Apart from this, Kaplan-Meier curve indicated that the HLH patients with post-treatment ferritin ≥1056.1 μg/L, platelets <100 × 10^9^ /L or ALT >40 U/L had significantly worse survival versus the patients with post-treatment ferritin <1056.1 μg/L, platelets ≥100 × 10^9^ /L or ALT ≤40 U/L (Fig. 1a-c).

### Establishment of clinical predict regression model

Based on the above three variables (ferritin, platelets and ALT), a prognostic model was developed to predict death within 6 months. The model showed that ferritin had the highest significant, followed by platelets and ALT. A higher count of platelets, and the lower value of ferritin and ALT in the model indicated the higher probability of survival in 6 months. The detailed coefficients as well as HRs were exhibited in Table 2. An equation that matches the model is established:

#### Model

$$ \mathrm{Logit}\ P=1.568\times \mathrm{platelets}-2.290\times \mathrm{ferritin}-0.860\times \mathrm{ALT}+3.150 $$

### Performance of model for risk of adult HLH patients’ prediction

**Fig. 2:**
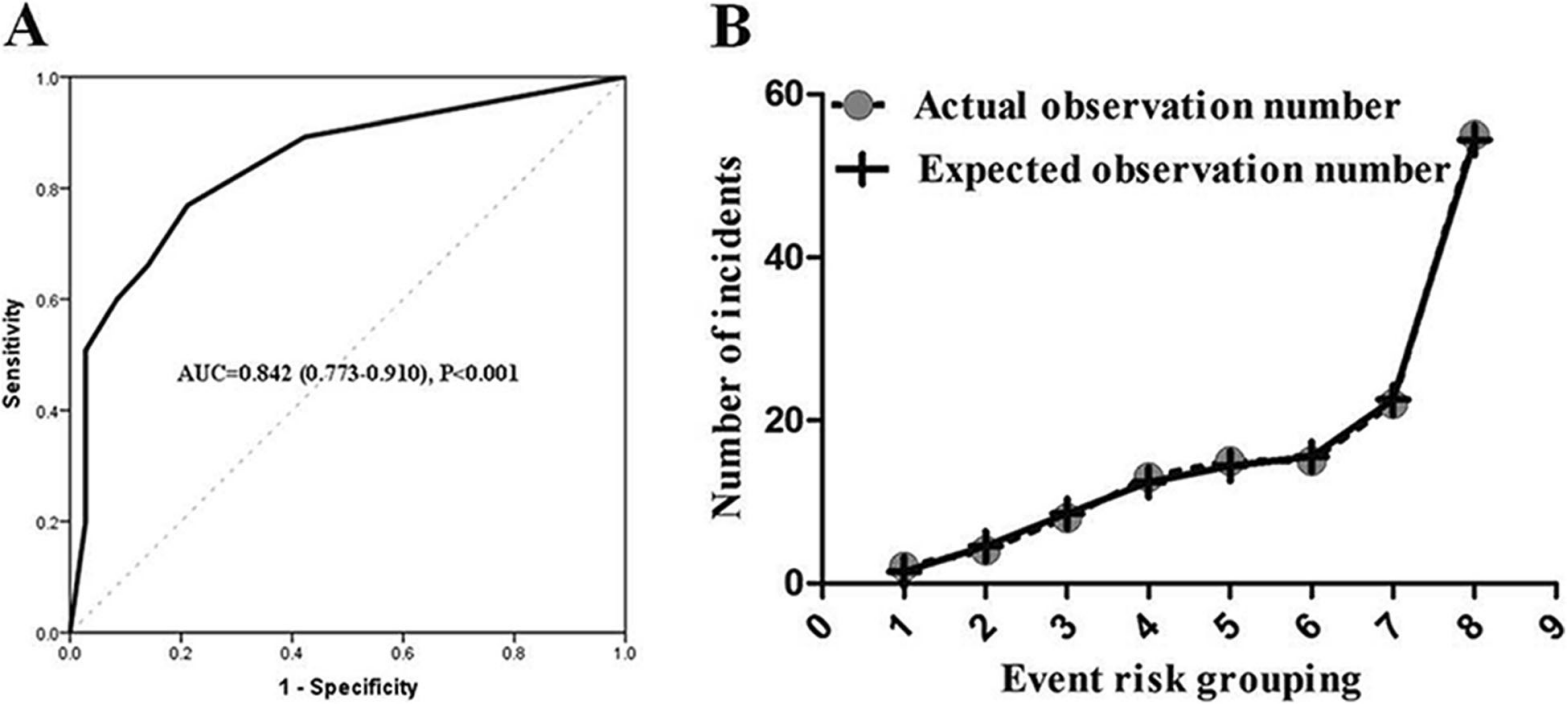
Performance of the model. **a** Characteristic feature of the curve for model. **b** Calibration curves for six-month OS, which are indicative of predictive accuracy

Receiver operating characteristic (ROC) was employed to compare the predictive values of mortality in adult HLH patients for the clinical model and independent prognostic adverse elements. The results showed that the area-under-the curve (AUC) values of the model was 0.842 (95% CI 0.773–0.910, *P* < 0.001) (Fig. 2), which was obviously superior than those of ferritin, platelets and ALT in cohort (ferritin: AUC 0.760, 95% CI 0.679–0.841, *P*<0.001; platelets: AUC 0.781, 95% CI 0.702–0.860, *P*<0.001; ALT: AUC 0.639, 95% CI 0.545–0.732, *P* = 0.005). We use sensitivity+ specificity- 1 to find the optimal cutoff value. Detailed values were showed in Table 3. In the model, 0.412 was selected as the best value to distinguish the low risk of death in HLH patients (sensitivity: 76.9%, specificity: 78.9%). We dichotomized the cohort based on the cutoff value of the model. The results showed that the patients with the value ≥0.412 had a markedly good prognosis while patients with value < 0.412 had a poor prognosis. Except for this, a significant difference was obtained between the two groups (*P* <0.001) (Fig. 1d).

**Table 3:** Sensitivity and specificity of the model at each cutoff point

Cutoff point	Sensitivity(%)	Specificity (%)	Youden’s index
0.249	89.2	57.7	0.469
0.412	76.9	78.9	0.558
0.583	66.2	85.9	0.521
0.685	60.0	91.5	0.515
0.764	50.8	97.2	0.480
0.867	33.8	97.2	0.310
0.933	20.0	97.2	0.172

The maximum value Youden’s index is the optimal value

Youden’s index = Sensitivity+ Specifcity- 1

## Discussion

In this study, we have developed a novel clinical prediction model to quantitatively identify the adult HLH patients at high risk of death within 6 months of diagnosis from a large retrospective cohort. We also validated that high ferritin and ALT act as adverse prognostic elements while high platelets play a positive role for survival in adult HLH.

Serum ferritin is engaged in acute phase response and can be found in all kinds of life [22–24]. Ferritin may exert pro-inflammatory effects by promoting the transcription of inflammatory mediators [25]. Zaher K et al. reported that there is a relationship between ferritin >50,000 mcg/L and 30-day mortality in adult patients with HLH [18]. Lin et al. showed that the pediatric HLH patients with ferritin levels decline ≥96% had a 17-time probability of survival when a comparison was made with ferritin levels fall <50% [26]. Similarly, our study also confirm that the higher post-treatment ferritin (≥1056.1 μg/L) is associated with higher odds of death in adult HLH patients, which is consistent with our previous study: post-treatment ferritin could be served as an prognostic factor in the adult HLH patients [27].

Platelets is a main bio-marker to assess one’s coagulation. Serum platelets levels vigorous decline could lead to abnormal coagulation. Zhao and his colleagues reported that platelets ≤39.5 × 10^9^ /L was an inferior prognostic factor in adult HLH [28]. Our results showed that platelets < 100× 10^9^/L should be considered as an independent six-month prognostic bio-marker in adult HLH which is consistent with previously published studies [29–32]. The potential mechanisms of thrombocytopenia in HLH may be due to destruction and consumption beyond the capacity of bone marrow regeneration. Bone marrow inhibition should not be ignored.

ALT is mainly found in hepatocytes and its elevation indicates damaged hepatocytes. Although ALT is not one of the eight diagnostic criteria for HLH, the hepatic function of adult HLH patients rang from gently elevation to full blown hepatic failure [33] (found in 83.6% HLH patients [15, 18]). Here, logistic regression shows that ALT could be a prognostic marker predicting probability of six-months survival with the AUC = 0.639, while combining with ferritin and platelets, the predictive efficiency is significantly improved.

In this study, based on three post-treatment serum markers (serum ferritin, platelets and ALT) extracted from 136 HLH patients, we developed a model to forecast the prognosis of HLH patients with all etiologies in six-month, which is different from one model for predicting the risk of 5-year overall survival in adult HLH patients not associated with malignancy. This forecast model, which was verified by ROC curves, had good sensitivity and specificity. Furthermore, the comparison between the model and each marker (ferritin, platelets and ALT) revealed that the performance of the model predicted six-month OS was obviously superior to single marker alone.Some limitations should be considered in our study. The impact of treatments on prognosis of patients were not analysis. We could not externally validate the results in sub-groups or verification group as HLH is a rare disease. Genetic measurement, Natural killer (NK) cell activity, sCD25, proinflammatory markers (IL-6, CRP), and coagulation index (D-dimer, antithrombin) were not included owing to lack of test results in this retrospective study. The lack of above data may be reason of low sensitivity and specificity of this model. A combination of this clinical variables and genetic markers might be able to improve the prognostic capability in HLH.

## Conclusions

In summary, we have established a laboratory based practical model from a large population-based cohort to prognosticate adult patients with HLH. This model was competent to discern patients who did survivor or not within 6 months. Additionally, for doctors, this model can be used to judge whether a patient met the discharge standards; for follow-up patients, the model can be used to determine a high-risk patient to be hospitalized in time.

## Methods

### Research subjects

Our investigation involved 136 newly diagnosed adult HLH patients (78 males and 58 females, aged 18–78, media age 50 year) at the hospital, from January 2010 to September 2018. The inclusion criteria were as follows: (1) Age ≥ 18y; (2) Newly diagnosis of HLH; (3) Meeting HLH-04 criteria 1) fever; 2) splenomegaly; 3) two or more cell lineage affected (HB < 90 g/L, platelets < 100 × 10^9^/L, neutrophils < 1.0 × 10^9^/L); 4) hypertriglyceridemia ≥3 mmol/L and/or fibrinogen (FIB) ≤1.5 g/L; 5) hemophagocytosis in the bone marrow, spleen, or lymph nodes; 6) low or absent NK cell activity; 7) ferritin ≥500 μg/L; 8) soluble interleukin-2 receptor levels ≥2400 U/mL); (4) Availability of relevant complete clinical data. Patients without follow-up results were beyond our scope.

### Research methods

The collected information of initial diagnosis as HLH was listed below: gender, age, clinical symptoms (pre-treatment) (pyrexia, hepatomegaly, splenomegaly, lymphadenopathy, rash, jaundice, edema, bone marrow hemophagocytosis) and laboratory data (pre- and post-treatment: ferritin, fibrinogen (FIB), neutrophils, HB, platelets, ALT, AST, LDH, α-HBDH, DBIL, triglycerides (TG), high density lipoprotein (HDL), low density lipoprotein (LDL), albumin, glucose, UREA, CREA, UA, Ca^2+^). There are two groups (survivor and non-survivor) based on the clinical follow-up evaluation at 6 months. The investigation was in line with the Declaration of Helsinki and authorized by the local institutional review board.

### Statistical analysis

Quantitative data, indicated as mean ± standard deviation (SD) or median (range), was compared by the independent sample *t* test between the two groups. Univariate and multivariate analyses were carried out to recognize the independent prognostic elements in adult HLH. All elements were filtered using the stepwise method by a multivariate binary logistic regression model. OS was defined as the time from the HLH initial diagnosis to the date of death from any cause or deadline of follow-up evaluation. The cutoff values from the predictive models were decided according to the maximum Youden index of the ROC curve. Kaplan-Meier was employed to evaluate survival probabilities. All above statistical analysis was performed using SPSS v21 software package. The predict potency of the forecast model was determined using a ROC curve with α = 0.05 as the statistically significant level and the comparison between the model and independent risk factors was done by MedCalc.

## Supplementary information

**Additional file 1: Table S1.** Pre-treatment clinical characteristics of patients according to outcome.

## Data Availability

The data and materials can be found from the corresponding author.

## Abbreviations

ALT: Alanine aminotransferase
AST: Aspartate aminotransferase
AUC: Area under the curve
Ca^2+^: Calcium
CI: Confidence interval
CREA: Creatinine
DBIL: Direct bilirubin
FIB: Fibrinogen
HB: Hemoglobin
α-HBDH: α-hydroxybutyrate dehydrogenase
HDL: High density lipoprotein
HLH: Hemophagocytic Lymphohistiocytosis
HR: Hazard ratio
NK: Natural killer
LDH: Lactate dehydrogenase
LDL: Low density lipoprotein
OS: Overall survival
ROC: Receiver operating characteristic
SD: Standard deviation
TG: Triglycerides
UREA: Urea nitrogen
UA: Uric acid
