# Supplementary material for: A novel prognostic model for adult patients with Hemophagocytic Lymphohistiocytosis
Source: Orphanet J Rare Dis. 2020 Aug 20;15:215. doi: 10.1186/s13023-020-01496-4 (PMC7439554; doi:10.1186/s13023-020-01496-4)
Supplement: Supplementary file 1 — Additional file 1: Table S1. Pre-treatment clinical characteristics of patients according to outcome. [file 13023_2020_1496_MOESM1_ESM.doc]

**Table S1.** Pre-treatment clinical characteristics of patients according to outcome

| Characteristics | Survivors, n = 65 | Nonsurvivors, n = 71 | P-value |
| --- | --- | --- | --- |
| **Gender (male/female), n** | 31/34 | 47/24 | 0.066 |
| **Median age (range), y** | 48(18-78) | 52(18-78) | 0.104 |
| **Fever ,n** | 62/65 | 68/71 | 0.913 |
| **Hepatomegaly** | 11/65 | 12/71 | 0.449 |
| **Splenomegaly** | 17/65 | 21/71 | 0.677 |
| **Lymph node enlargement** | 11/65 | 14/71 | 0.653 |
| **Rash** | 9/65 | 8/71 | 0.640 |
| **Jaundice** | 14/65 | 13/71 | 0.596 |
| **Edema** | 8/65 | 11/71 | 0.725 |
| **Bone marrow emophagocytosis** | 49/65 | 56/71 | 0.444 |
| **Ferritin≥500(μg/L)** | 58/65 | 64/67 | 0.224 |
| **FIB (g/L)** | 2.75 ± 1.46 | 2.42 ±1.20 | 0.192 |
| **Neutrophils (×10^9^/L)** | 4.11 ± 3.87 | 3.74 ±5.38 | 0.657 |
| **HB (g/L)** | 100.35 ±23.86 | 95.98 ±23.60 | 0.288 |
| **Platelet (×10^9^/L)** | 78.37 ±61.09 | 73.58 ±71.42 | 0.678 |
| **ALT (U/L)** | 123.42 ±148.77 | 180.54 ±377.50 | 0.260 |
| **AST (U/L)** | 202.47 ±263.55 | 283.81±598.25 | 0.319 |
| **LDH (U/L)** | 971.84 ±1022.66 | 1129.77 ±1691.46 | 0.523 |
| **α-HBDH (U/L)** | 586.56 ±673.51 | 663.60 ±724.71 | 0.536 |
| **DBIL (μmol/L)** | 13.07 ±17.60 | 33.32 ±61.19 | 0.013 |
| **TG (mmol/L)** | 2.22 ±1.28 | 2.93 ±5.66 | 0.329 |
| **HDL (mmol/L)** | 0.66 ±0.32 | 0.88 ±2.18 | 0.426 |
| **LDL (mmol/L)** | 2.05 ±0.76 | 2.59 ±3.42 | 0.223 |
| **Albumin (g/L)** | 30.70 ±6.10 | 28.21 ±5.56 | 0.017 |
| **Glucose (mmol/L)** | 7.23 ±6.07 | 5.88 ±1.83 | 0.092 |
| **UREA (mmol/L)** | 4.72±1.87 | 8.16 ±7.78 | 0.001 |
| **CREA (μmol/L)** | 57.07±20.37 | 91.07±110.85 | 0.016 |
| **UA (μmol/L)** | 239.33±134.91 | 291.60±184.81 | 0.071 |
| **Ca^2+^ (mmol/L)** | 2.02±0.16 | 2.00±0.18 | 0.586 |
